# The magnitude of ivacaftor effects on fluid secretion via R117H-CFTR channels: Human *in vivo* measurements

**DOI:** 10.1371/journal.pone.0175486

**Published:** 2017-04-18

**Authors:** Jessica E. Char, Colleen Dunn, Zoe Davies, Carlos Milla, Richard B. Moss, Jeffrey J. Wine

**Affiliations:** 1 Cystic Fibrosis Research Laboratory, Stanford University, Stanford, California, United States of America; 2 Department of Pediatrics, Stanford University School of Medicine, Stanford, California, United States of America; University Medical Center Utrecht, NETHERLANDS

## Abstract

We optically measured effects of orally available ivacaftor (Kalydeco®) on sweat rates of identified glands in 3 R117H subjects, each having a unique set of additional mutations, and compared them with 5 healthy control subjects tested contemporaneously. We injected β-adrenergic agonists intradermally to stimulate CFTR-dependent ‘C-sweat’ and methacholine to stimulate ‘M-sweat’, which persists in CF subjects. We focused on an R117H-7T/F508del subject who produced quantifiable C-sweat off ivacaftor and was available for 1 blinded, 3 off ivacaftor, and 3 on ivacaftor tests, allowing us to estimate *in vivo* fold-increase in sweat rates produced by ivacaftor’s effect on the open probability (P_O_) of R117H-CFTR. Measured sweat rates must be corrected for sweat losses. With estimated sweat losses of 0.023 to 0.08 nl·gland^-1^·min^-1^, ivacaftor increased the average C-sweat rates 3–7 fold, and estimated function as % of WT were 4.1–12% off ivacaftor and 21.9–32% on ivacaftor (larger values reflect increased loss estimates). Based on single tests, an R117H-7T/ R117H-7T subject showed 6–9% WT function off ivacaftor and 28–43% on ivacaftor. Repeat testing of an R117H-5T/F508del subject detected only trace responding to ivacaftor. We conclude that *in vivo*, R117H P_O_ is strongly increased by ivacaftor, but channel number, mainly determined by variable deletion of exon 10, has a marked influence on outcomes.

## Introduction

Cystic fibrosis (CF) is caused by mutations in CFTR anion channels. The membrane conductance mediated by CFTR (G_CFTR_) is equal to the number of functional channels in the membrane (n), their average open probability (*P*_*O*_), and their single channel conductance (γ): (G_CFTR_ = n*P*_*O*_γ). Some mutations reduce n and others decrease *P*_*O*_ or γ. The resulting decreases in G_CFTR_ reduce epithelial anion-mediated fluid secretion in organs such as sweat glands, lungs, intestines and pancreas, and most CF symptoms follow from these defects. Ivacaftor *in vitro* increases *P*_*O*_ in CFTR channels having a wide variety of gating mutations [1, 2], and patients with some of these mutations show marked clinical improvement when treated with oral ivacaftor (ivacaftor) [3–6].

The R117H mutation has complex effects, and our understanding of these effects is improving. Early work showed R117H to have mild effects on gating and conductance (*P*_*O*_γ) that reduced function in whole cell experiments to 15% of normal [7–9]. More recent work, which used strategies to avoid underestimating the number of channels in a patch, concluded that γ is reduced by 25% and P_O_ is reduced 13-fold [10], while other work showed R117H also to be a mild folding mutation that reduces channel number [11]. The combined defects should reduce function to <15% of WT, or <7.5% when paired with a nonfunctional mutation such as F508del. Nevertheless, most subjects with R117H/F508del do not have typical CF symptoms—they are most commonly encountered when screening CBAVD patients [12]. Thus, CF disease occurs when R117H CFTR channel function is decreased still further. The most common situation in which this occurs is when R117H is in *cis* with a 5T polythymidine allele, causing increased missplicing that results in ~10% full-length transcripts [13]. If the other allele is a nonfunctional mutation such as F508del, the higher end estimated CFTR function relative to healthy controls will be the product of the 5T (~10%), R117H (~15%) and one non-functional allele (50%) giving 0.75% predicted WT function [14]. Remarkably, such subjects are pancreatic sufficient, consistent with other observations that very small amounts of CFTR function can ameliorate CF disease severity [14, 15].

Ivacaftor (VX-770) increased R117H-CFTR as measured via ion transport [8, 9, 11], or the P_O_ of patch-clamp recorded R117H-CFTR channels [10], and ivacaftor improved several clinical parameters including sweat chloride values in CF subjects [16]. The goal of the present study was to quantify effects of ivacaftor on R117H function *in vivo* using a new sweat rate bioassay for CFTR function in individually identified sweat glands [14, 17]. The standard sweat chloride assay induces sweating via pilocarpine iontophoresis and then measures the concentration of chloride in the collected sweat [18]. The sweat chloride assay is very sensitive to changes in the lowest levels of CFTR function, but becomes less sensitive as CFTR function increases [14]. The sweat rate assay complements the sweat chloride assay by providing a near-linear readout of CFTR function over most of its range, so that, for example, carriers can be distinguished from WT subjects. However, it is less sensitive at very low levels of CFTR function such as seen with R117H-5T. Therefore, we were eager to apply the method to two subjects with R117H on a 7T background, where increased channel number produced sufficient function to measure sweat rates off as well as on ivacaftor for most of their glands. Because it measures multiple, identified glands in parallel, the assay is particularly well suited for n-of-1 studies.

In the present study, we used an n-of-1 paradigm to estimate ivacaftor’s effect on CFTR *P*_*O*_ in a subject with genotype R117H-7T/F508del. The 7T allele is expected to produce a much milder phenotype, and indeed, almost 50% of this subject’s glands secreted measureable amounts of C-sweat off ivacaftor (vs. ~zero amounts in R117H-5T subjects). This enabled us to compare CFTR function (±) ivacaftor and to estimate how oral dosing with ivacaftor increased the *P*_*O*_ of the R117H-CFTR channels in the cells of the secretory coils of this subject’s sweat glands. We also tested one R117H-7T/R117H-7T subject with single, unpaired tests in the off and on conditions. To provide context we studied one R117H-5T/F508del subject and 5 healthy controls. In addition to examining ivacaftor’s effects, the experiments help to refine some aspects of the methodology, and incorporated new information from *in vitro* studies [8–11] to help calibrate the assay.

## Materials and methods

### Subjects

The study was approved by the Institutional Review Board of Stanford University. After written informed consent, 8 subjects were studied: 5 healthy controls and 3 subjects with R117H mutations. The main study focused on one adult male with genotype R117H-7T/F508del who was participating in a multi-center clinical trial of oral ivacaftor (ivacaftor) [16]. At the completion of the blinded portion of that study, we recruited this subject (coded S9) for an independent, n-of-1 study of CFTR-dependent and independent sweat secretion during his washout and open label phases. S9 is an adult male subject who is infertile, has FEV_1_ values 50–60% predicted, a history of non-mucoid *Pseudomonas aeruginosa* or *Staphylococcus aureus* lung infections, but normal sweat chloride of ~20. We tested him seven times: once in the blinded condition (after his last blinded study visit), three times in the off drug period and three times in the open label. In addition to the main study, we performed single tests on and off ivacaftor for an R117H-7T/R117H-7T subject who was included in the study. For comparison with these 7T subjects, we tested a -5T subject (R117H-5T/F508del) with a series of 8 tests: 3 off drug, 2 on drug, and 3 blind (later shown to be placebo). Five healthy control subjects were also tested without drug. There were no adverse events.

### Reagents

Methacholine Chloride, (Methapharm, Ontario, Canada), Isoproterenol HCl, Aminophylline, lactated Ringer’s (Hospira, Lake Forest, IL) and Atropine Sulfate, (American Reagent) were obtained from Stanford University Hospital Pharmacy. Heavy mineral oil was from EMD Chemicals, Gibbstown, NJ, and was water-saturated before use as previously described [17]. Erioglaucine disodium salt (CAS No. 3844-45-9) was from Sigma. Ivacaftor (Kalydeco®) was provided to subjects by Vertex as part of a clinical trial [16].

### Ratiometric measurement of sweat secretion from identified individual glands

We used a modified version of the single gland, ratiometric, optical imaging assay for CFTR secretory function [17]. The assay depends on two parallel pathways for sweat secretion: a CFTR-independent, a cholinergic pathway stimulated with methacholine that persists in CF and is considered to be CFTR-independent (‘M-sweat’) and a β-adrenergic pathway that is CFTR-dependent (‘C-sweat’) [17]. When C-sweating is expressed as a function of M-sweating, it provides a near-linear readout of CFTR function over a wide range, e.g. the C-sweat/M-sweat ratio for CF carriers is 50% that of non-CF controls and the ratio for CF subjects is zero [17, 19]. In brief, a specific, identified region of skin on the volar forearm was sequentially injected intradermally with methacholine to stimulate CFTR-independent sweating (M-sweat) and then with a cocktail of isoproterenol, aminophylline, and atropine in lactated Ringer’s to block M-sweating and produce C-sweating.

Conditions used in the present assay were modified in 3 ways. First, M-sweat was stimulated with .05 ml of a 1 μM solution of methacholine, which is 1/2 the volume used previously. Second, M-sweat was monitored for only 10 min, instead of the 15 min used in prior tests [14, 17]. The lower amount and shorter duration of methacholine had the advantage that merging of M-sweat bubbles was reduced, and pilot tests indicated that it still potentiated C-sweating as reported previously. Third, C-sweating was monitored for 30 min as before, but because of a miscommunication the studies were begun using only 0.05 ml of cocktail instead of the 0.1 ml used previously [17]. We kept this level of stimulation throughout this study. Because the agonist levels were changed the results in this study can’t be directly compared with prior ones—therefore a set of contemporaneous control subjects were run with the new concentrations for comparisons.

Bubbles of sweat from single glands were captured in an oil layer, visualized by oblique lighting or dye-partitioning, and digitally imaged at 30 sec intervals. Individual glands were identified by location relative to landmarks and one another (gland constellations). For each identified gland the increases in M- and C-sweat volumes over time were recorded and average M- and C-sweat rates/min were calculated by dividing the final sweat volumes for each gland by 10 or 30 min respectively. In the plots of M- and C-sweat correlations, each point represents the average rate for a single identified gland based on all trials where the gland’s secretion was measured.

### Loss minimization and loss correction

Several factors limit the accuracy of sweat gland secretory readout of CFTR function. The premise of this assay is that the primary C-sweat produced by the secretory coil provides a linear readout of CFTR function if gland size is controlled by measuring M-sweat: if CFTR function is 0, 50 or 100% of WT, so too will be the C/M ratio [17, 19]. However, we do not measure primary sweat, but instead measure conditioned sweat after it has traversed the reabsorbtive duct, where some of it is lost because of physical capacitance (the duct needs to fill) and because of some ductal absorption [17]. While we know we need a loss factor we can only approximate its magnitude, and lacking a better model we are using a constant loss factor. The effect of a constant loss of C-sweat increases proportionally as the C-sweat rate slows, eventually exceeding the C-sweat rate in glands with low residual CFTR function. We previously estimated the constant loss to be 0.023 nl·min^-1^·gl^-1^ or ~2% of the average WT C-sweat value [14], and used this estimate to explain the inability of the imaging assay to detect sweating in most glands of most subjects with PS CF. Here, we reexamine the consequences of different constant loss estimates with regard to estimates of *P*_*O*_ increases produced by ivacaftor, and use information from in vitro studies [8–11] to help us calibrate the sweat loss.

Comparisons among averaged ratios can be done in two ways: by determining each gland’s C/M ratio for the on and off conditions separately and then averaging those ratios (the mean of the ratios), or by taking the ratio of the average C and M responses in each condition (the ratio of the means). We found good agreement between the two methods, so for simplicity we report only the ratio of the means.

### Statistical analysis

Single, identified sweat glands were the units of analysis for the within-subject comparisons in this “n-of-1” analyses [17]. Paired t-tests of original or log transformed data, Pearson r, and ANOVA were used as appropriate.

## Results

### Subject S9, R117H-7T/F508del

**Fig 1:**
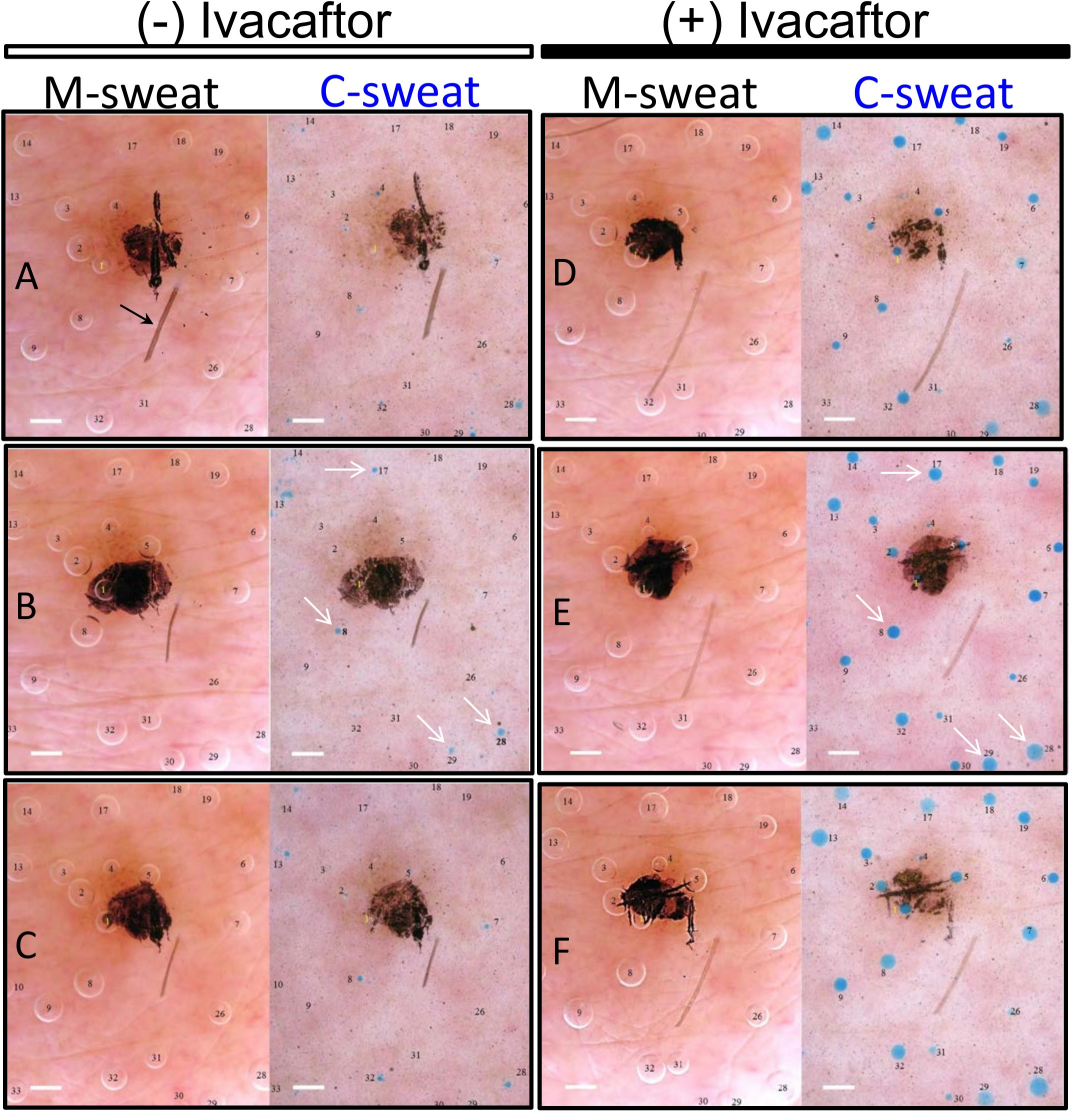
Methacholine-stimulated (M-sweat) and CFTR-dependent (C-sweat) bubbles imaged in an oil layer on the forearm of an R117H-7T/F508del subject off and on ivacaftor. Each image shows a mid-region (~22 mm^2^) of the stimulated field of sweat glands (full imaged field is ~63 mm^2^). Results are shown for 6 tests, A-C off ivacaftor and D-F on ivacaftor. Each pair of images shows ~ 18 M-sweat bubbles, each produced by a single, identified sweat gland. M-sweat (clear bubbles) accumulated during 10 min of methacholine stimulation. C-sweat (blue bubbles) accumulated during 30 min of stimulation with a β-adrenergic cocktail that included atropine to block M-sweat; this test directly followed the methacholine test at the same site. Blue dye particles dispersed in the oil during the C-sweat trials stained the bubbles blue to improve their visibility [17]. A freckle (light brown spot) and a hair (arrow, panel A) provided natural landmarks. A spot of ink placed on the freckle improved focusing and registration between M- and C-sweat trials. Calibration bar = 0.5 mm.

S9, an infertile adult male with FEV_1_ values <60% predicted, bacterial lung infections, but normal sweat chloride values of ~20 mM, had markedly reduced C-sweat rates that were dramatically increased by oral dosing with ivacaftor. **Fig 1**shows 6 pairs of images of the same central portion of the field for subject S9. Each image shows 20 identified glands that were followed across 7 trials (6 are shown here) over a ~ two month period; the image pairs are arranged in chronological order from earliest (**Fig 1A**) to most recent (**Fig 1F**). A clipped hair (arrow) is a landmark across all trials; a new ink spot is added at the start of each trial. M-sweat bubbles are unstained and C-sweat bubbles are stained blue. **Fig 1A–1C**show results from the three off ivacaftor tests. Seven of 20 glands in this field produced small but quantifiable C-sweat on at least one trial off ivacaftor; 4 of the most prominent are labeled (arrows) in Fig 1B and 1E. **Fig 1D–1F**show results from the three tests on ivacaftor. All 20 glands in this field produced measureable C-sweat on at least one trial on ivacaftor and the volumes were much greater. The M-sweat bubbles volumes varied little across trials, in contrast with the marked increase in C-sweat bubble volumes on ivacaftor.

**Fig 2:**
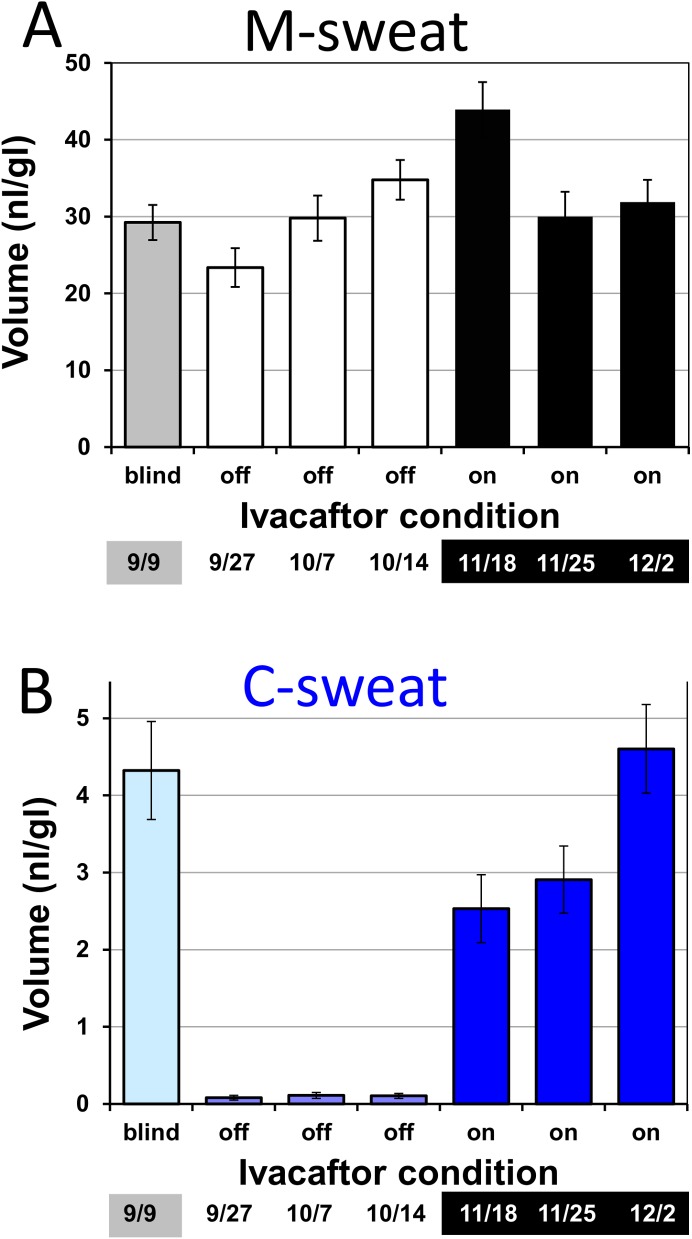
Average volumes of secreted sweat as a function of stimulus and drug for R117H-7T/F508del subject S9 (A) Bar graphs showing mean ± SEM of accumulated M-sweat volumes per gland ± ivacaftor; 3 tests in each condition and in one blinded test, based on a 10 min monitoring period. (B) Bar graphs showing accumulated C-sweat volumes per gland ± ivacaftor; 3 tests in each condition and one blinded test, based on a 30 min monitoring period. Each bar graph represents mean ± SEM of 38–41 glands.

The average results across all 7 trials are graphed by date and condition in **Fig 2**. Average M-sweat volumes (**Fig 2A,** overall mean rate = 2.13 ± 0.42 nl·min^-1^·gl^-1^) did not differ significantly across ivacaftor conditions, ANOVA, P = 0.4, but gland-by-gland analysis did reveal a small difference (see below). By contrast, average C-sweat volumes/gland increased dramatically in the on ivacaftor condition **(Fig 2B)**. The total volumes of measured C-sweat for 38 glands x 3 trials were 10.9 nl off ivacaftor and 380 nl on ivacaftor, or an average of 3.6 nl/trial off ivacaftor and 126.7 nl/trial on ivacaftor (P < 0.01, ANOVA). The mean C-sweat rate in picoliters·min^-1^·gl^-1^ was 3.2 ± 0.8 off ivacaftor *vs*. 111.1 ± 18.3 on ivacaftor. **Fig 2B** suggested that the subject was receiving ivacaftor during the blinded test (C-sweat rate of 142.9 ± 25.3 pl·min^-1^·gl^-1^), and this was subsequently verified when the trial [16] was unblinded.

**Fig 3:**
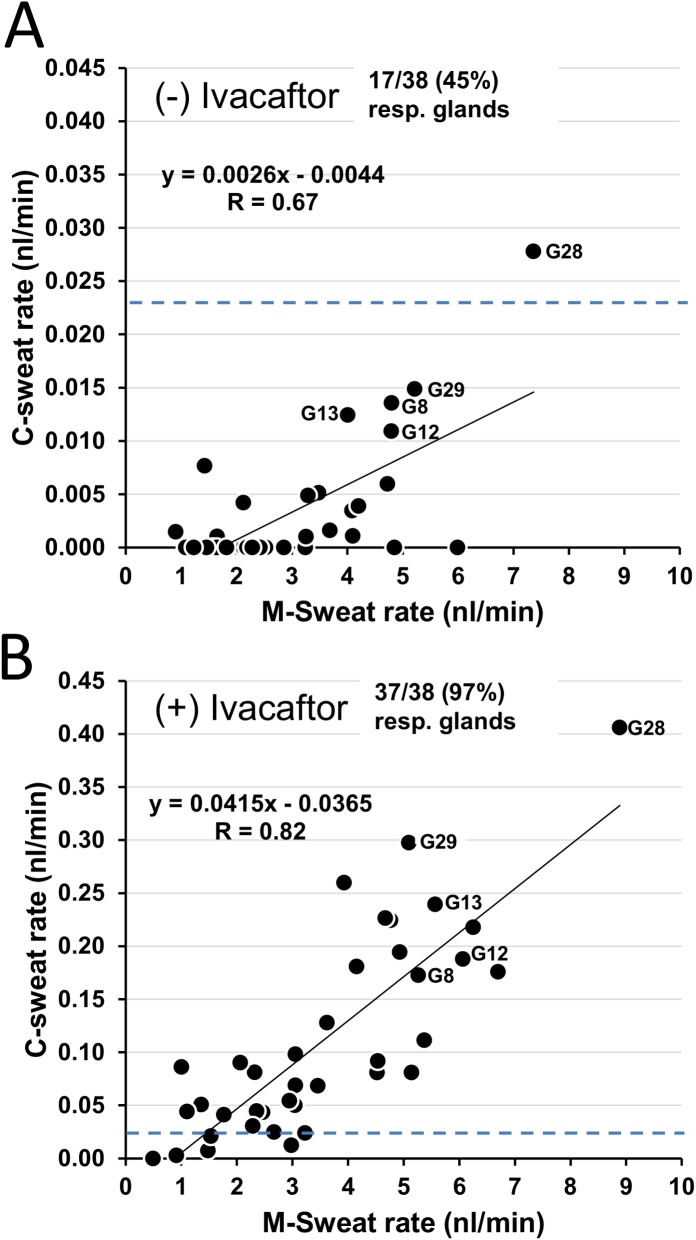
Subject S9 (R117H-7T/F508del): gland-by-gland sweat secretion (±) ivacaftor. Each point is jointly determined by the mean of M-sweat rates (x-axis) and C-sweat rates (y-axis). (A) off ivacaftor, (B) on ivacaftor; y-axis is 10X the axis of A. n = 38 glands. Five glands with the highest C-sweat rates off ivacaftor are labeled. Data were not corrected for sweat-losses; the estimated loss of C-sweat (0.023 nl·min^-1^·gl^-1^) is indicated by the dashed horizontal lines.

Sweat rates are plotted on a gland-by-gland basis for all 38 identified glands in **Fig 3**. Each point is jointly determined by the average M- and C-sweat rates for that gland off ivacaftor (**Fig 3A)** and on ivacaftor (**Fig 3B)**. Five glands with the highest C-sweat rates off ivacaftor are labeled in both graphs. The response rate (defined as measurable secretion on at least 1 of 3 trials) increased from 17/38 glands (45%) off ivacaftor to 37/38 glands (97%) on ivacaftor, and the slope of C- vs. M-sweat increased markedly. These data were graphed without correcting for sweat losses; the dashed horizontal lines indicate the sweat loss estimate of 0.023 nl·min^-1^·gl^-1^. Uncorrected data for S9 and all other subjects is summarized in **Table 1**.

**Table 1:** Summary data: R117H and WT subjects, all data, uncorrected.

				Gland numbers			Secretion rates		Ratios	
ID	G	Iva. off or on	Trials(n)	MCh Glands (n)	Cktl Glands (n)	C/M n glands (%)	M-sweat Rate (nl/min/gl)	C-sweat Rate (nl/gl/min)	C mean/ M mean	% WT (see text)
**F508del / R117H-7T see 001-summary tab from DST with corrected data.xlsx**										
**S9**	**M**	blind	1	38	32	84%	3.0 ± 1.5	0.14 ± .12	0.047	29.4%
		**–**	**3**	**38**	**10±1**	**26±3%**	**2.9 ± 1.5**	**.0032 ± .0058**	**0.001**	**0.68%**
		**+**	**3**	**38**	**34±3**	**90±7%**	**3.5 ± 0.8**	**0.111 ± .095**	**0.032**	**19.5%**
**F508del / R117H-5T**										
**S10**	**M**	**–**	**3**	**44.5**	**0.25**	**0.2%**	**2.95 ± 1.1**	**0**	**0**	**~0%**
		**+**	**2**	**46**	**0**	**0.0%**	**2.75 ± 0.4**	**0**	**0**	**>0%**
**R117H-7T / R117H-7T (note: off and on tests carried out on *different* glands)**										
**S11**	**M**	**–**	**1**	**40**	**26**	**65%**	**5.33 ± 0.67**	**.033 ± .010**	**0.006**	**3.0%**
		**+**	**1**	**54**	**53**	**98%**	**9.75 ± 0.83**	**0.54 ± 59**	**0.054**	**27.0%**
**Healthy Controls**										
**WT05**	**M**	-	2	49	49	100%	4.9 ± 0.26	1.00 ± 0.07	0.20.2	126%
*WT10	**F**	-	3	51	49.6	98%	2.1 ± 1.0	0.32 ± 0.02	0.15.1	94%
**WT11**	**F**	-	1	53	48	91%	2.5 ± 1.2	0.40 ± 04	0.15.6	98%
**WT13**	**M**	-	3	37	37	100%	5.1 ± 0.17	0.84 ± 05	0.16.5	103%
**WT14**	**F**	-	3	60	60	100%	2.9 ± 0.13	0.36 ± 0.02	0.12.5	78%
**Mean±SD**		-	**2.4**	**50±8**	**49±8**	**98.1%**	**2.7 ± 1.5**	**0.52 ± 0.27**	**0.16 ± .03**	**100%**

**Uncorrected summary for all data from 3 R117H and 5 WT subjects.** Table 1 is arranged by subject and condition. Each row shows the mean results for the number of trials performed. For R117H subjects the top row for each subject shows results off ivacaftor (or blind) and the bottom row on ivacaftor. Columns labeled ‘gland numbers’ show the number of identified glands that secreted M-sweat to an intradermal injection of MCh: ‘MCh Glands’, then CFTR-dependent sweat to a β-adrenergic cocktail at the same site: ‘Cktl Glands’, and the ratio of the two expressed as a percentage. Columns labeled ‘secretion rates’ show the average M- or C-sweat rates (per min, per gland). M-sweat rates are based on a 10 min observation period; C-sweat rates on a 30 min period. Subjects had been taking ivacaftor for at least 3 weeks prior to on ivacaftor testing. Columns labeled ‘ratios’ show the ratio of C-sweat mean rate/M-sweat mean rate, and the percentage of this ratio to the mean ratio for the 5 WT subjects. *Data for WT10 were reported in a previous study [14].

### Estimating the magnitude of ivacaftor effects on fluid secretion via R117H-CFTR

The presence of measureable secretion rates in 17 of the 38 glands off ivacaftor provided an opportunity to estimate the fold increase of the fluid secretion increase produced by oral ivacaftor on R117H-CFTR. CFTR is rate-limiting for C-sweat [19], and R117H is mainly a gating mutation [7, 10, 20]. Although recent data indicate that R117H is also results in misfolding and reduced channel number, there is no evidence that R117H CFTR channel number changes as a result of ivacaftor treatment. Single channel data indicate that γ is also unchanged by ivacaftor; the sole ivacaftor effect being a 2–4 fold increase in R117H CFTR *P*_*O*_ [9, 10]. When R117H CFTR was stably expressed in FRT cells and tested with a gradient, ivacaftor produced a 4.1 fold increase in Cl^-^ transport [8], and when expressed in CFBE41o- cells VX-770 produced a 2.4 fold increase [11].

**Fig 4:**
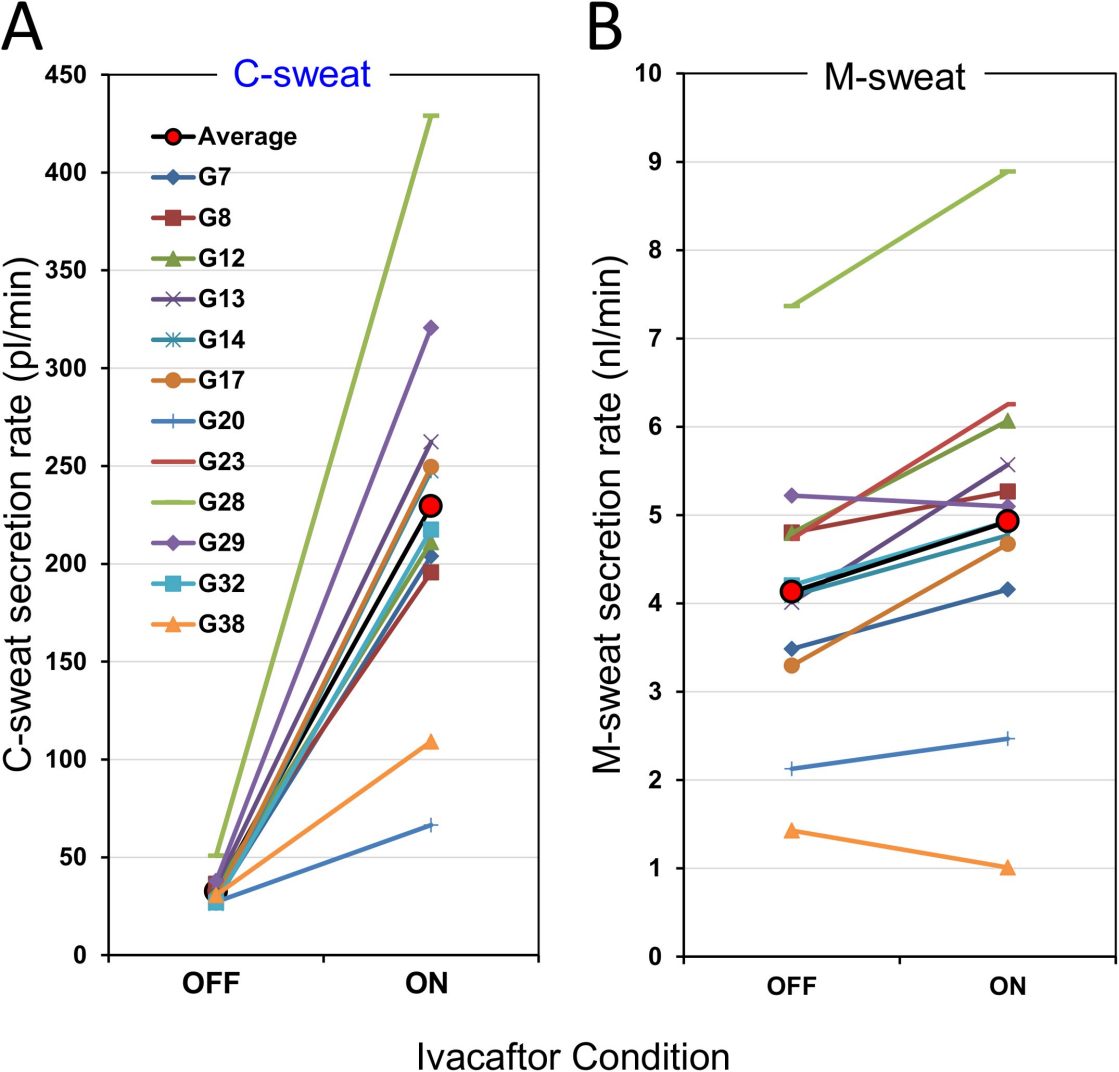
Magnitude of ivacaftor-induced increases in M and C-sweat secretion for the top 12 glands of R117H-7T/F508del subject S9 Data for the 12 glands with the largest mean C-sweat rates off ivacaftor. (A) C-sweat rate increased markedly as a function of ivacaftor treatment, (P = 6.58E-09 two tailed paired t-test on logged data). (B) M-sweat increased slightly for the same glands (P = 0.02 two tailed t-test on logged data). Each point is an identified gland’s average secretion rate for 3 off and 3 on tests. Values were corrected for sweat volume losses by adding 0.023 nl/min to all M- and C-sweat rates (see text).

To estimate the fold increase in C-sweat rates we used the 12 glands with the highest C-sweat rates off ivacaftor (because these are proportionately less affected by sweat loss) and applied the previously determined loss correction of 0.023 nl·min^-1^·gl^-1^ [14]. The results are plotted in **Fig 4A**. The mean loss-corrected rates off/on ivacaftor were 32.6 ± 7 pl·min^-1^ and 229 ± 88 pl·min^-1^, a 7 ± 2 -fold increase (P = 8.1E-06, paired t-test). This increase is ~1.7–3 times the fold increases observed with *in vitro* measurements [8–11]. Even given the different conditions, the agreement between these estimates seems too large to be within experimental error, suggesting that the loss factor estimate may be too low (see discussion). Loss-corrected data for S9 and all other subjects is summarized in **Table 2**.

**Table 2:** Summary data: R117H and WT subjects, top 12 glands; loss-corrected.

ID	G	ivacaftoroff or on	Trials (n)	M*-sweat rate	C*-sweatRate	C* mean/ M* mean	C* mean/ M* mean % WT
**R117H-7T / F508del**							
**S9** **7T**	**M**	**-**	3	4.15 ± 0.44	0.03 ± .002	0.008	4.1%
		**+**	3	4.95 ± 0.56	0.21 ± .027	0.042	21.9%
**R117H-5T / F508del**							
**S10 5T**	**M**	**-**	3	4.43 ± 0.4	0	0	0
		**+**	2	3.95 ± 0.3	~0	~0	~0
**R117H-7T / R117H-7T**							
**S11** **7T/7T**	**M**	**-**	1	10.44 ± 0.99	0.11 ± 0.02	0.011	6.1%
		**+**	1	18.51 ± 2.03	0.92 ± 0.16	0.05	27.7%
**Healthy Controls**							
**WT-05**	**M**	**-**	2	6.9 ± 0.36	1.66 ± 0.09	0.243	127%
**WT10**	**F**	**-**	3	3.4 ± 0.2	0.6 ± 0.025	0.178	93%
**WT11**	**F**	**-**	1	4.1 ± 0.2	0.84 ± 0.07	0.203	106%
**WT13**	**M**	**-**	3	6.7 ± 0.4	1.24 ± 0.06	0.184	96%
**WT14**	**F**	**-**	3	4.0 ± 0.27	0.61 ± 0.023	0.152	79%
**WT av.**	**-**		**2.4**	**5.02 ±1.65**	**0.99 ± 0.21**	**0.192 ± .01**	**100%**

**Loss-corrected summary data for top 12 glands from the subjects shown in Table 1.** Except for omitting the ‘gland numbers columns’ (n = 12 glands were selected from each subject) the row and column categories prior to ratios columns are the same as Table 1. The 12 glands were selected based on C-sweat rates in the off ivacaftor condition except for S10, where M-sweat rates were used since no glands produced C-sweat off ivacaftor. For each subject the same glands were then compared across conditions.

### Evidence for an ivacaftor effect on M-sweat rates

In previous work with G551D subjects we had equivocal evidence that ivacaftor might increase M-sweating [14]. Gross comparisons in the present experiments (Fig 2A) showed no significant difference in M-sweating (±) ivacaftor in this R117H-7T subject, but paired comparisons of individual glands, which provides a more powerful means to detect small differences, showed a small and marginally significant increase in M-sweat rates on ivacaftor the same 12 glands selected for analysis of C-sweat rates. The mean rate increased from 4.1 to 4.9 nanoliter·min^-1^gland^-1^ (P<0.02, paired t-test on logged data, **Fig 4B**).

### Ivacaftor effect on C-sweat in S11, a subject homozygous for R117H-7T

**Fig 5:**
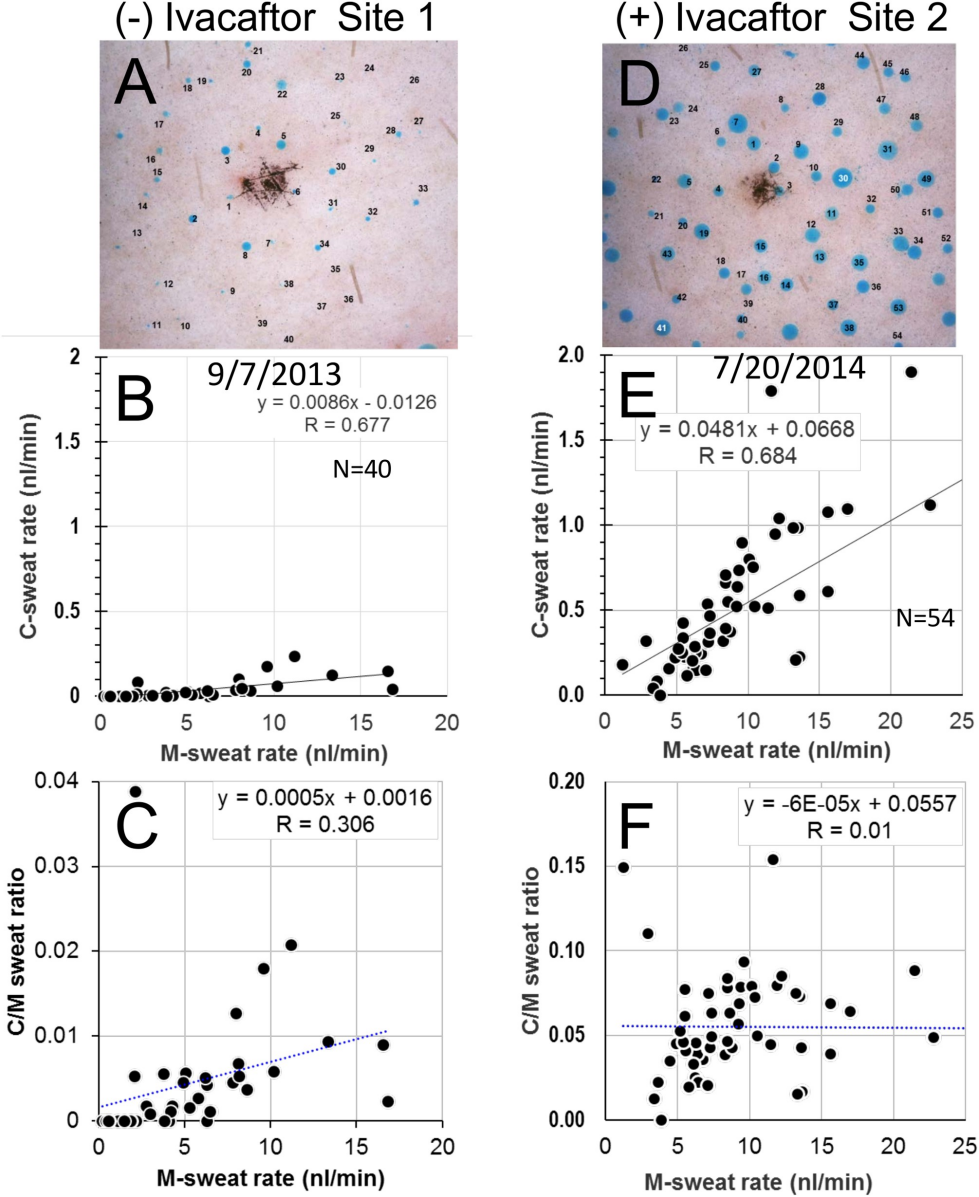
Subject S11 (R117H-7T/R117H-7T). S11 was tested once each at two different sites. (A-C) Responses off ivacaftor at site 1. (D-E) Responses on ivacaftor at site 2. (A, D) C-sweat bubbles off/on ivacaftor at 30 min time point. (B, E) C-sweat rate vs. M-sweat rate off/on ivacaftor. Each point represents a single test for a single gland and shows its M-sweat rate on the x-axis and its C-sweat rates on the y-axis. (C, F) C/M ratios vs. M-sweat rates off/ on ivacaftor. Each point represents a single test for a single gland and shows its M-sweat rate on the x-axis and its C/M sweat ratio on the y-axis. Note that y-axis scale for (F) is 5X that of (C).

We had limited access to a single R117H-7T/R117H-7T CBAVD subject (**S11**). **S11** completed only one test off and one test on ivacaftor separated by 10 months; the subject started ivacaftor the day of his first off test and was on drug the entire time up to the on test. The tests inadvertently measured glands at 2 different sites several cm apart on the same arm, precluding gland-by-gland comparisons. In spite of these limitations, we saw clear evidence for abnormally low C-sweating off ivacaftor and a large effect of ivacaftor. **Fig 5A and 5D**shows the C-sweat responses (uncorrected) at the two sites and two conditions. The C-sweat rate as a function of M-sweat rate at site 1 off ivacaftor (**Fig 5B**) was far below normal; only 23/40 (58%) glands produced measureable C-sweat vs. control average of 98%. It was dramatically larger on ivacaftor at site 2 (**Fig 5E**), where 53/54 glands produced C-sweat.

Analysis of the C/M ratio as a function of M-sweat rate across the two sites and conditions (**Fig 5C and 5F**) showed two effects: a marked increase over all (note different y-axis scales) and a reduced slope of the regression line which occurs as the loss factor becomes less significant at higher secretion rates. The increased C/M ratio at site 2 on ivacaftor occurred in spite of an almost a 2-fold higher M-sweat rate seen at site 2 on ivacaftor (**Table 1**). Although comparison across sites is not ideal, we previously compared multiple sites within one subject and found highly reproducible C-sweat/M-sweat ratios across the sites [17], so we are confident that the increased C/M ratio resulted from oral ivacaftor dosing and not site differences. (No other sweat test methodology requires identical sites.) The data shown in Fig 5 were not corrected, corrected summary data is in Table 2.

### S10, an R117H-5T/F508del subject, had near-zero C-sweat responses

**Fig 6:**
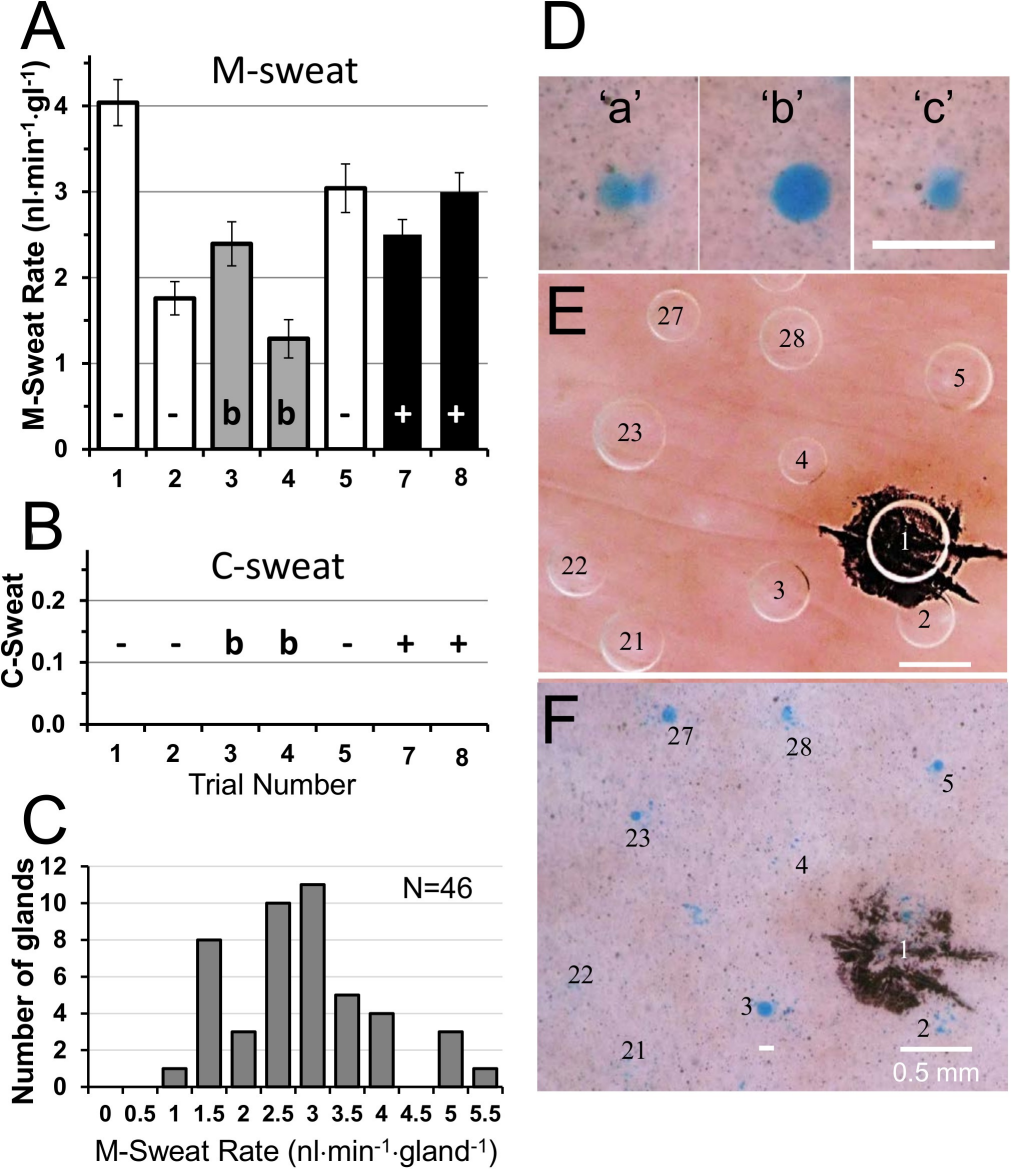
S10 (R117H-5T/F508del) had near-zero C-sweat responses. (A) M-sweat rates across 3 off trials (open columns) 2 blind trials (gray columns,) and 2 open-label on ivacaftor trials (black columns). (B) The corresponding C-sweat rates for 46 glands in the ROI were zero across all trials. (C) Distribution of average M-sweat rates. M-sweat rates for each gland were averaged across 7 trials and the rate distribution plotted to illustrate that the lack of C-sweating does not result from unusually low M-sweat rates. (D) Examples of 3 glands that secreted on ivacaftor on trial 7; ‘b’ was the largest C-sweat bubble seen for this subject. Three different glands produced measureable C-sweat on trial 8 but were outside the ROI. (E) M-sweat bubbles at a different site (left arm) used on trial 6 on ivacaftor because the standard site was obstructed by an IV line. (F) Small C-sweat bubbles were observed for 4/35 glands at this site, but no corresponding testing was done off ivacaftor. Calibration = 0.5 mm for D, E, F.

**S10** (F508del/R117H-5T) is a pancreatic sufficient CF subject who was tested 8 times: 2 blinded, and 3 each off and on ivacaftor. Unblinding of the clinical trial [16] after the sweat rate analysis revealed that both blinded tests were off ivacaftor. M-sweating showed significant variation across trials unrelated to ivacaftor (**Fig 6A**). **Fig 6B** shows that no verifiable C-sweating occurred within the region of interest (ROI), which was empirically determined as the more central region where sweat bubbles could be measured most clearly. Because smaller glands secrete more slowly, we checked the distribution of M-sweat rates (**Fig 6C**) and saw that 8 glands secreted M-sweat at average rates ≥4 nl·min^-1^·gl^-1^, well above rates for glands that produced consistent C-sweat on ivacaftor in an R117H-5T subject studied previously [14]. Yet no C-sweat responses were seen even from **S10**’s fastest secreting glands. Several glands outside of the ROI did respond (**Fig 6D**), and a different site for which no control data are available was tested once on ivacaftor (Test 6), and at this site 4/46 (9%) glands produced very small responses (**Fig 6E and 6F**). In sum, this pancreatic sufficient subject produced a very small amount of C-sweat on ivacaftor, but only in regions where we did not have definitive evidence of zero sweating off ivacaftor. Because trace amounts of sweating sometimes occur in PS subjects, we do not claim that an ivacaftor effect has been established for **S10**.

### Responses of control subjects and comparisons with R117H subjects

**Fig 7:**
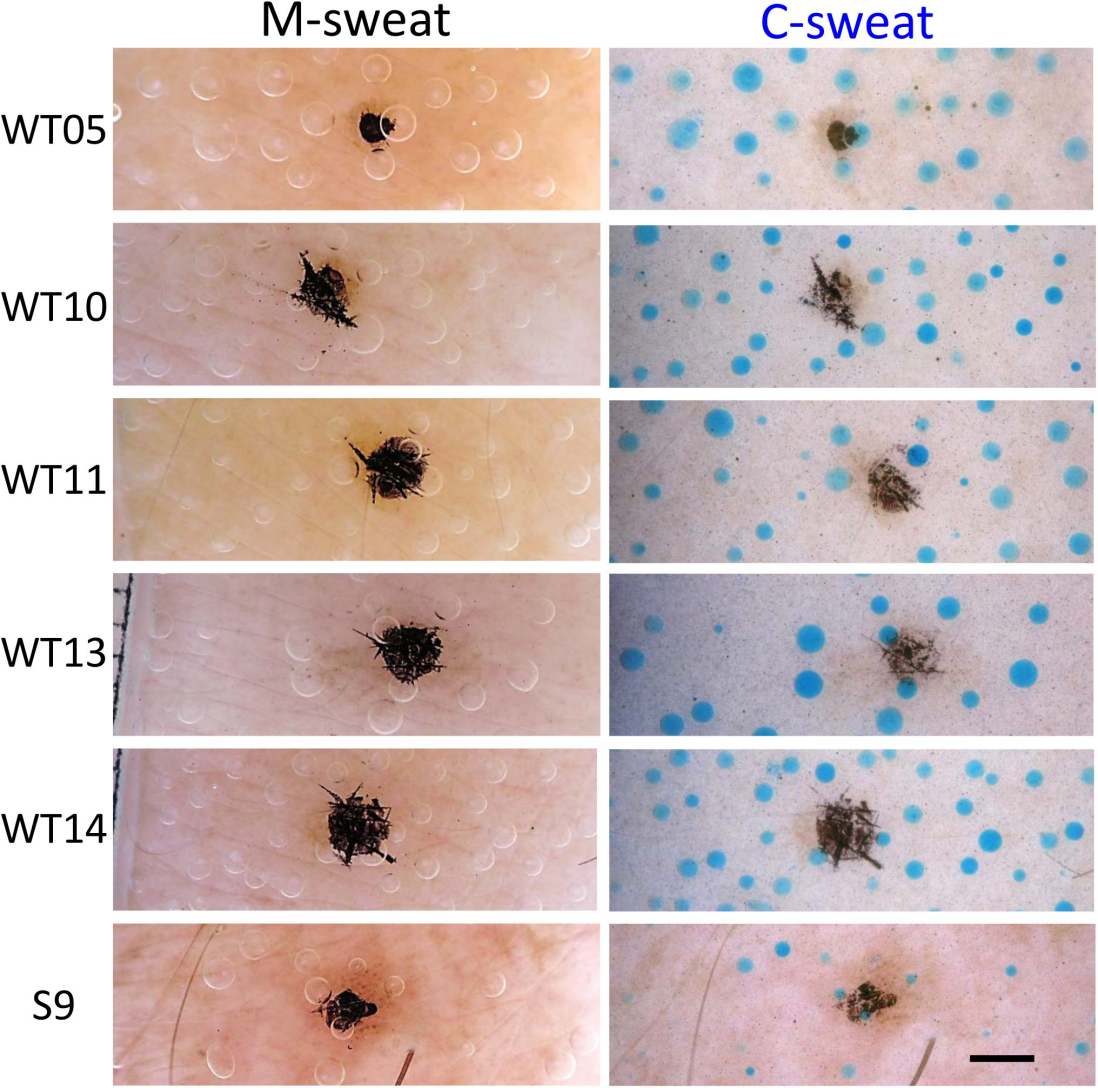
M-sweat (left column) and C-sweat (right column) bubbles for five control subjects and the R117H-7T/F508del subject S9. The image for S9 is from one blinded test we had access to from his clinical trial [16] with ivacaftor. Images were cropped from the center of the field. Scale bar = 1 mm.

We tested 5 control subjects using identical conditions to provide contemporaneous control data. On average, 50 identified glands per subject were measured over 1–3 assays and were used to generate the data in **Tables 1 and 2.** Examples of M- and C-sweat sweat bubbles for the control subjects are shown in **Fig 7**along with images from the one blinded trial with **S9**, the R117H-7T/F508del subject. After the trial concluded and was unblinded it was revealed that S9 was receiving ivacaftor at the time of the blinded test (see Figs 1 and 2B).

**Fig 8:**
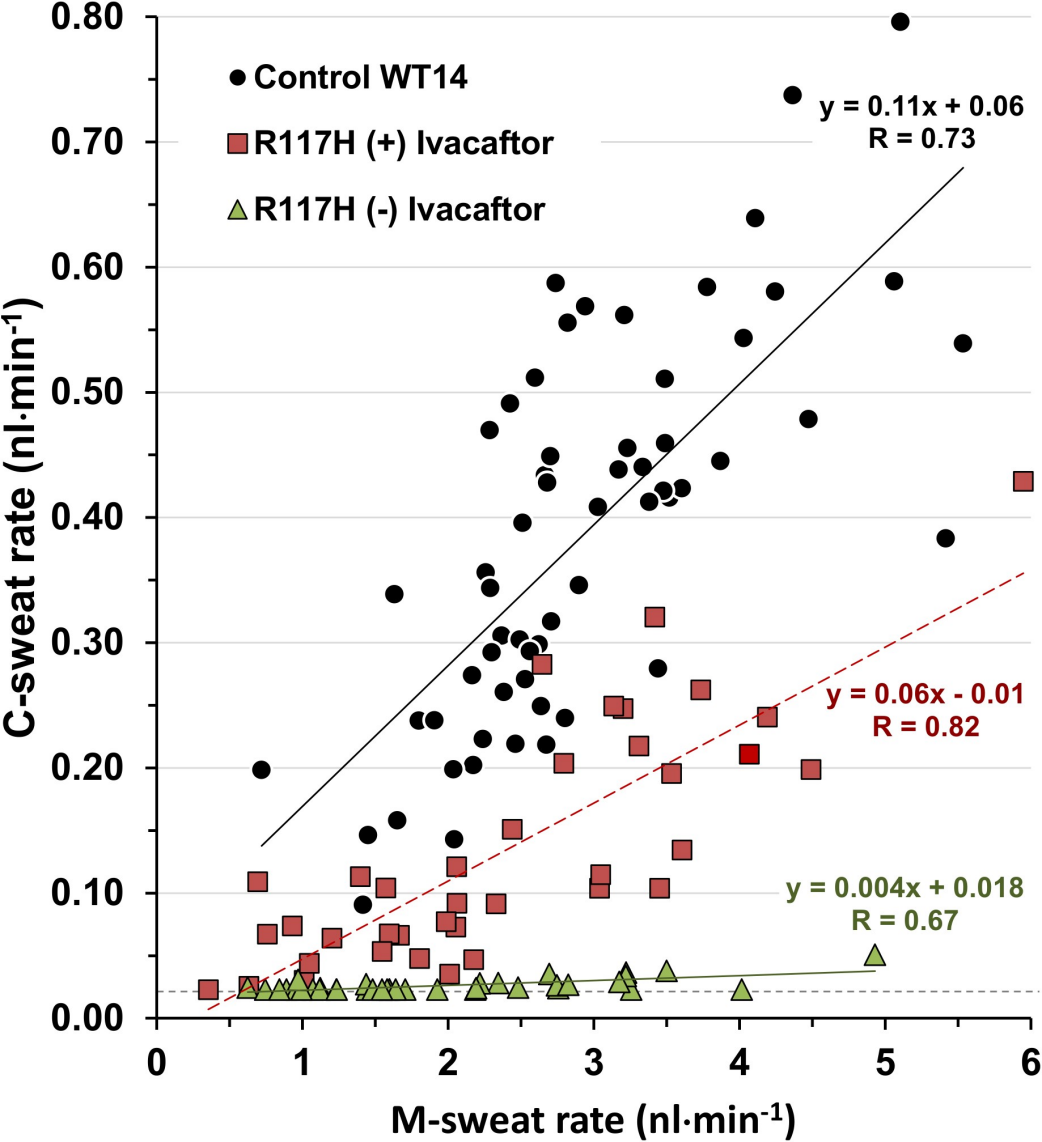
Gland-by-gland comparisons of a control subject (WT14) with the R117H-7T subject (±) ivacaftor. Each point is jointly determined by the mean M-sweat rate on x-axis and the mean C-sweat rate on the y-axis for a single gland across 3 trials; n = 60 glands for WT14 and n = 38 glands for R117H-7T. All data were loss-corrected by adding 0.023 nl·min^-1^ to the rates; this imposes a minimum rate, indicated by the dashed horizontal line, for glands with no visible sweating.

A gland-by-gland comparison between S9 (±) ivacaftor and one control subject (WT14) is shown in **Fig 8**, where each point was jointly determined by the mean C-sweat and M-sweat responses for that gland across 3 trials. WT14 had the lowest C-sweat response of the 5 control subjects. WT14 and S9 had similar M-sweat rate distributions (x axis), but S9 had a markedly lower C-sweat response in the absence of ivacaftor (triangles). After ivacaftor, (squares) almost every gland showed increased responding with the largest absolute changes occurring in the glands with higher M-sweat rates.

**Fig 9:**
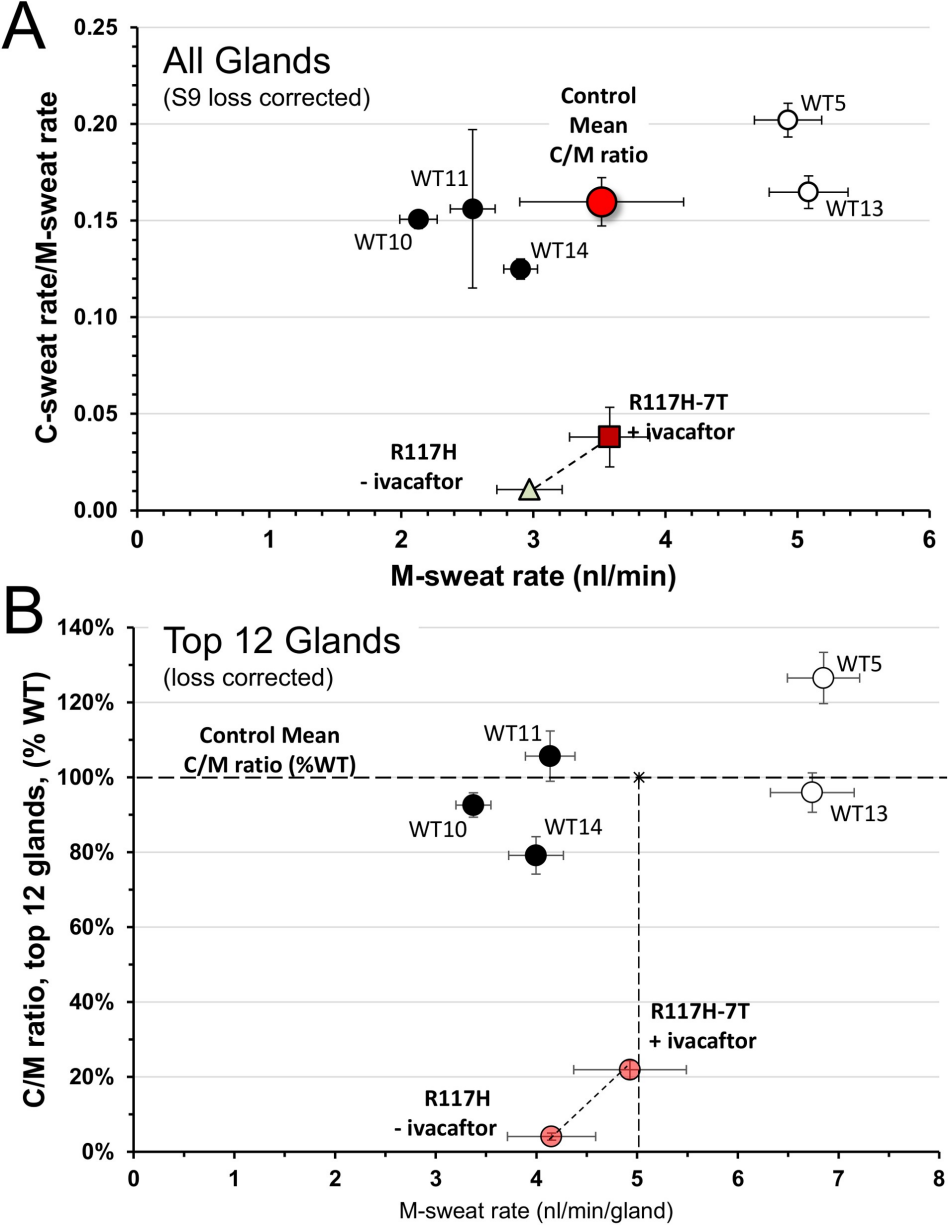
Mean C/M ratios vs M-sweat rates for 5 control subjects and S9, R117H-7T (+/-) ivacaftor. (A) Average C/M sweat rate ratios for all glands. Each symbol plots the mean M-sweat rate on the x-axis and C/M-sweat rate ratio on the y-axis for a single subject, and represents 37–60 glands and 1–3 tests per WT subject (Table 1). Filled circles: WT females; open circles: WT males. WT mean is also plotted. Data for the R117H-7T subject is the mean of 38 glands (+/-) ivacaftor across 3 trials in each condition. Ratios for the R117H subject were based on loss-corrected data. (B) Average C/M sweat rates for top 12 glands, loss corrected, and expressed as % WT average. Each symbol plots for a single subject the mean M-sweat rate on the x-axis and C/M-sweat rate ratio as a % of the WT average on the y-axis. Each symbol is the mean of the 12 glands for that subject with the highest average C-sweat rates across 1–3 tests (see Table 2). WT C/M mean is shown as dashed horizontal line. The 12 glands for S9 (R117H-7T/F508del) were those having the highest C-sweat rates off ivacaftor.

Summary data for the 5 controls and for S9 (+/-) ivacaftor is presented in **Fig 9**. **Fig 9A** presents the mean ratios of C/M sweat rates for all measured glands from each of the control subjects and for S9 (R117H-7T/F508del) off/on ivacaftor. Using data from all of the glands, ivacaftor increased both C- and M-sweat as shown. **Fig 9B** is an alternative approach to comparing the data. For each subject we averaged data from the 12 glands with the highest C-sweat rates, which reduces the contribution of the loss factor and hence should give a more accurate estimate of CFTR function. To provide meaning to the C/M ratio, we normalized the data by setting the average WT C/M ratio for these 5 subjects = 100% (indicated by the dashed line) and expressed each subject’s data as a percentage of the average.

## Discussion

The main goal of this study was to quantify changes in R117H-CFTR mediated sweat gland fluid secretion produced by oral ivacaftor. This was possible because of the availability of an R117H-7T/F508del subject who was available for multiple measurements on and off ivacaftor. Unlike R117H-5T subjects, R117H-7T subjects should have near-normal numbers of R117H-CFTR transcripts [13, 21–24], although channel number is reduced because R117H also displays a folding defect [11]. S9 displayed a level of C-sweat in the absence of ivacaftor that was sufficient to allow the ivacaftor effect to be meaningfully expressed as fold-increases in C-sweat. We attribute the increase to *P*_*O*_ because ivacaftor increases *P*_*O*,_ but not channel number or γ [9, 10]. The precision of the estimate was increased by identifying individual glands and tracking those glands across 3 trials with and 3 without ivacaftor. Precision was further increased by selecting the fastest secreting glands where the loss factor is proportionally less, and by adding back the estimated loss. Using these methods, we estimated that ivacaftor produced a 7 ± 2 fold increase of C-sweat and a 20% increase of M-sweat (Fig 4B), and increased this subject’s CFTR function from 4.1% to 21.9% of WT function (Table 2). A second R117H-7T homozygote, tested less stringently, had corresponding figures of 6.1% and 27.7% WT function, while for an R117H-5T subject we observed essentially zero C-sweat off drug and only trace amounts on ivacaftor (Table 2).

**Fig 10:**
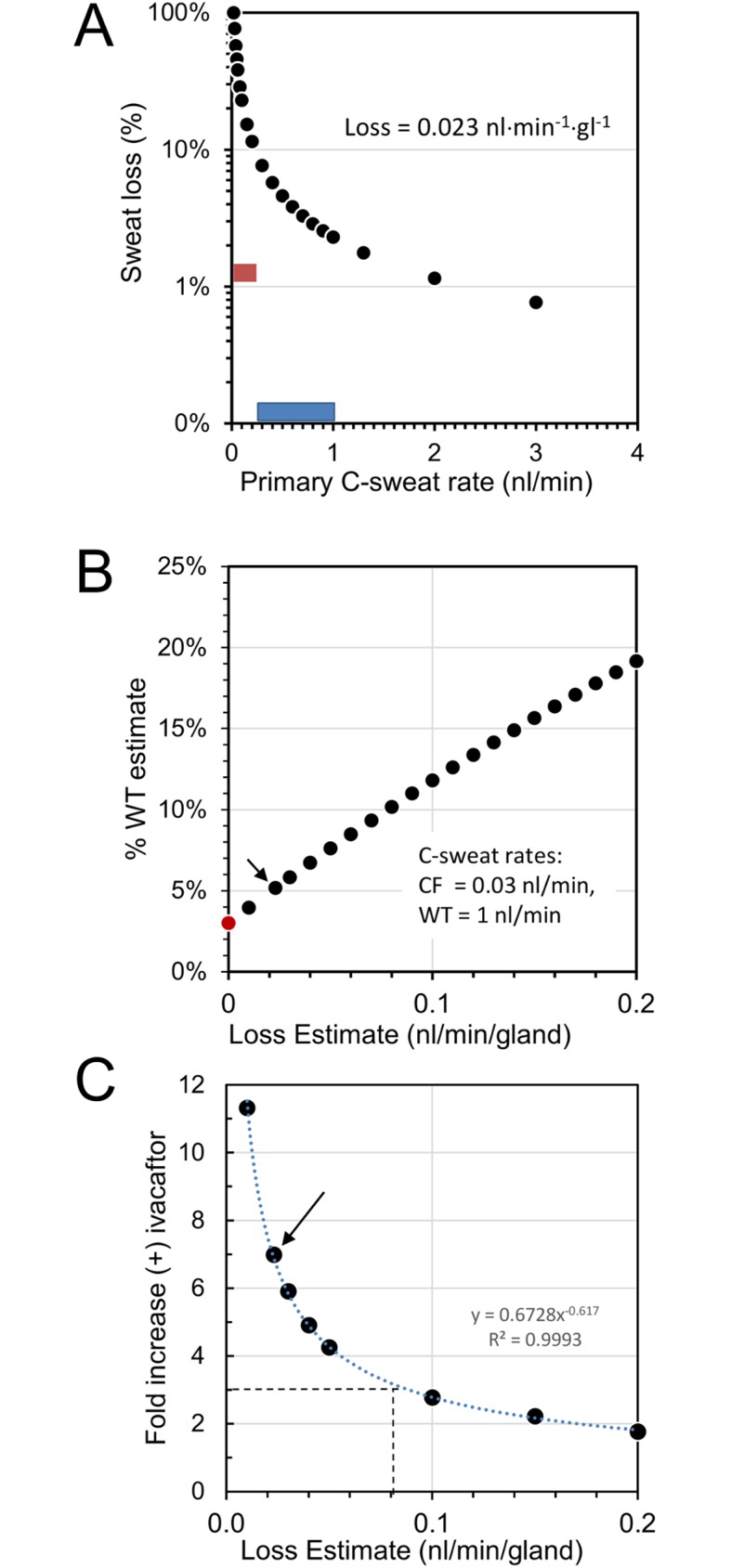
Interactions between sweat rates, sweat losses and estimates of CFTR function. (A) The **%** sweat lost decreases as sweat rate increases. The % of sweat that is lost is plotted on the y axis, a log scale, vs. the primary C-sweat rate on the x axis. This is for a constant loss of 0.023 nl·min^-1^·gl^-1^. The red bar shows the range of individual gland C-sweat response rates for the R117H subjects (Table 1). The horizontal blue bar shows the range of mean C-sweat responses for the 5 control subjects used in this study (Table 2). (B) Plots of the consequences of different loss estimates (x-axis) on the % WT estimate (y axis) for an observed C-sweat rate = 3% of WT rate (red circle), and various loss-corrected values and corresponding %WT estimates as black circles. The 0.023 nl/min estimate (arrow) corrected the rate to ~5% of WT. (C) Plots of the effect of different loss estimates on the fold-increase in C-sweat rate observed with ivacaftor. A loss estimate of 0.023 nl·min^-1^·gl^-1^, corresponds to a fold increase of 7 (arrow), while a loss estimate of 0.08 nl·min^-1^·gl^-1^ corresponds to a fold-increase of 3 [10].

As we have noted [14, 17], the %WT estimates are influenced by loss estimates. The loss estimate used here was based on previous measurements of an R117H-5T subject who had an extremely low level of C-sweat off ivacaftor [14]. The much higher rates in R117H-7T subject S9 provide another opportunity calibrate the loss factor and to quantify how it affects estimates of function. **Fig 10**illustrates the interdependencies among observed sweat rate, sweat losses, and estimates of CFTR function. As plotted in **Fig 10A**, for a fixed loss the proportion of sweat lost increases as sweat rate decreases. With a loss estimate of 0.023 nl·min^-1^·gl^-1^ the loss rises steeply in the range of C-sweat rates seen with R117H mutations (red bar). **Fig 10B** shows that as the loss estimate increases so too does expression of the defective sweat rate as a percent of the WT rate. **Fig 10C** shows how increasing loss estimates produce decreases in the calculated fold-increases in C-sweat rate observed on ivacaftor. For S9 the loss estimate of 0.023 nl·min^-1^·gl^-1^, gives a fold increase of 7 ± 2 (arrow). To better calibrate the loss factor we turned to *in vitro* results.

### Comparisons of *in vitro* and *in vivo* estimates of mutated CFTR function

**Fig 11:**
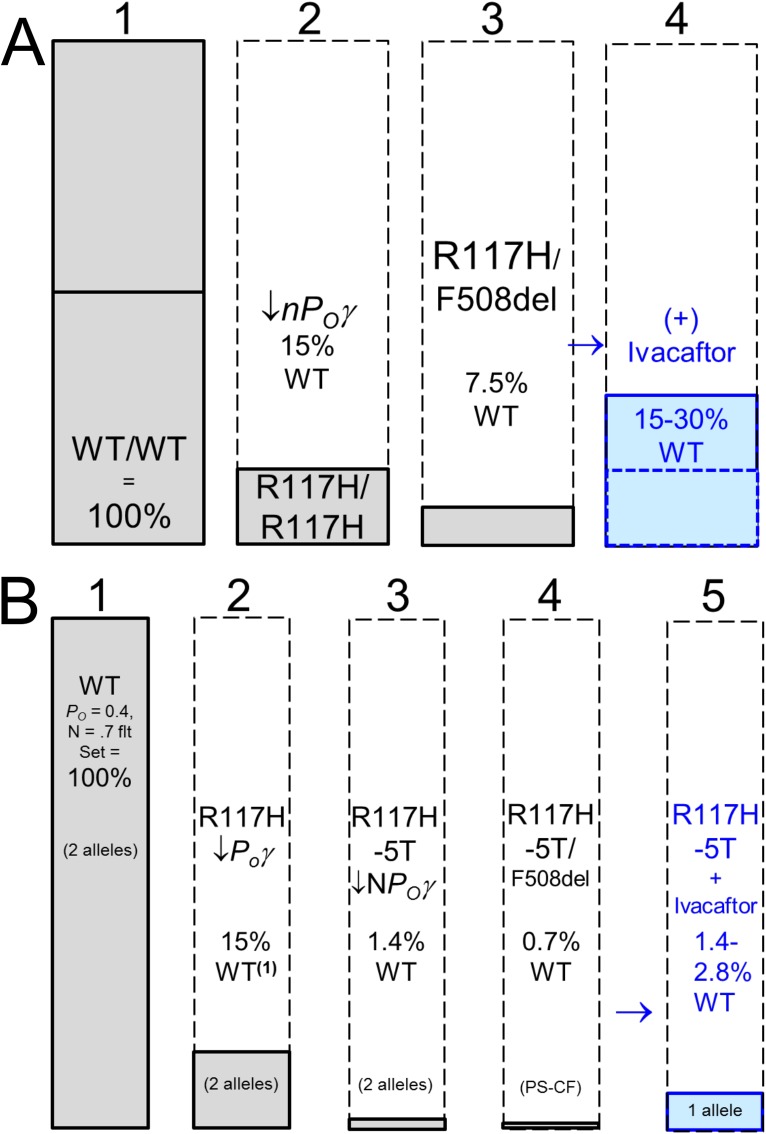
R117H-7T and R117H-5T function (+/-) ivacaftor: estimates from *in vitro* measurements of channel function and transcript analysis. Each column indicates the proportion of CFTR function remaining for the conditions shown based on in vitro measurements of channel function and transcript analysis. (A) R117H-7T with reduced n*P*_*O*_γ to ~15% WT (column 2) produces CBAVD [25]. Pairing with a non-functional allele (column 3) predicts ~7.5% WT function—such subjects rarely present at CF clinics. Ivacaftor increases *P*_*O*_ 2–4 fold to give 15% (dashed line) to 30% WT function. (B) R117H-5T. The 5T mutation causes a large decrease in full-length transcripts and when combined with R117H reduces function to ~1.5% (column 3) and to 0.75% when the other allele is non-functional (column 4). If ivacaftor increases Po 2–4 fold it should provide ~1.4–2.8% WT function to subjects with an R117H-5T/non-functional allele.

Data from *in vitro* experiments with R117H and VX-770 can be used to cross check the sweat rate estimates. What % WT function is predicted for R117H based on *in vitro* experiments? As mentioned in the introduction, recent patch clamp experiments conclude that R117H P_O_γ is about 6% of WT [10], and in a surprising finding, Gentzsch and colleagues [11] showed that R117H is also a folding mutation with reduced channel number. Based on correction by VX809 (their Fig 1D), it appears n could be reduced to ~70% of WT. The combined defects would lower function to ~4% of WT, and if the other allele is nonfunctional it would predict ~2% function. Earlier whole-cell experiments of Sheppard *et al*. [7], which would reflect both reduced P_O_γ and n, gave a value of 15% WT, or 7.5% function if the other allele is nonfunctional. Thus in vitro experiments indicate R117H function to be between 4–15% of WT or 2–7.5% if the other allele is non-functional. We estimated 4.1% function for S9 (R117H-7T/F508del), and 6.1% function for S11 (R117H-7T/R117H-7T). These are in the lower range of values predicted by the in vitro experiments, suggesting that the loss factor we used is an underestimate. The highest value of 15% WT was used in **Fig 11A**, which shows the predicted level of CFTR function for an R117H-7T/F508del subject like S9 to be 7.5% of WT.

A second method for cross-checking *in vivo* and *in vitro* results compares the level of correction produced by VX-770 *in vitro* with that produced by ivacaftor *in vivo*. Multiple *in vitro* experiments using various methodologies have shown increases of 2–4 fold [8–11], whereas for S9 we calculated an average increase of 7-fold (range 2.4–8.4 for individual glands) in the 12 highest secreting glands after a loss correction of 0.023 nl·min^-1^·gl^-1^ (Fig 4A). As shown in Fig 10C, the fold-increase will decrease as the loss estimate increases. The middle *in vitro* estimate of a 3-fold increase [10] conforms to a loss estimate of 0.08 nl·min^-1^·gl^-1^ (Fig 10C, dashed lines). If we use this loss estimate, it increases the calculated %WT function for S9 to 12% off ivacaftor and 32% on ivacaftor, values higher than highest *in vitro* estimates.

A complication in trying to compare fold-increases of channel P_O_ with sweat rates is that changes in CFTR *P*_*O*_ might be amplified by the recruitment of Cl^-^ transport mechanisms. When apical Cl^-^ channels open, efflux of Cl^-^ lowers [Cl^-^]_i_ resulting in phosphorylation and activation of the basolateral Na+-K+-2Cl- co-transporter NKCC1 [26, 27] via WNK, SPAK and OSR1 pathways [28–30]. It is unknown how the loss of Cl^-^ activates the WNK pathway and how that is related to agonist-triggered events. Ivacaftor may provide a novel way to study these relations, since in our studies the level of agonist stimulation is identical in the cells off and on ivacaftor, with the only difference being a change in *P*_*O*_ of the CFTR channels. (It is an important simplification that when C-sweat is produced purely by β-adrenergic stimulation no basolateral K^+^ channels are activated) [31]). This hypothesis could be tested by studying VX-770 effects on R117H-mediate transport in secretory epithelia that are not permeabilized to allow normal basolateral mechanisms to operate.

### Other R117H subjects

We also tested S11, R117H-7T homozygote who also showed a large response to ivacaftor, and S10, R117H-5T/F508del, where essentially no C-sweat was observed before or after ivacaftor. We hypothesize that CFTR in the sweat gland secretory coil cells of all R117H subjects responded to ivacaftor with the same increases in *P*_*O*_, and attribute the different C-sweat rates among them to different numbers of CFTR channels expressed in their sweat glands and to the need for C-sweat to reach ~2% of WT before it can be measured. R117H-5T subjects only express ~10% full length CFTR transcripts [13, 21–24], and CFTR n is also affected by TG repeats [32], and its function by the M470V allele [32, 33]. **Fig 11B** shows the expected response levels for some configurations of R117H-5T, using the highest *in vitro* estimate of 15% WT function for R117H. The majority of R117H subjects seen in CF clinics are R117H-5T/severe allele, for which *in vitro* results predict function that is <1% of WT and thus below this assay’s resolution.

### Does CFTR contribute to M-sweating?

An unexpected finding from this study was evidence that CFTR contributes to M-sweating. M-sweating has been considered CFTR-independent because it persists and can be robust in people with CF [18, 34]. However, in multiple experiments that distinguished between β-adrenergic and cholinergically-stimulated sweating, somewhat reduced cholinergic sweating has been seen for CF subjects [17, 19, 35], although it was not seen in a more recent, well-designed evaporimetry study with a large cohort of subjects [36]. CFTR might contribute to M-sweating because (1) CFTR contributes to cholinergically-mediated fluid secretion in several tissues and species [37–43]; (2) stimulation of M3 muscarinic receptors strongly activates CFTR when both are expressed in BHK cells [44], (3) apical UTP activates CFTR in primary human airway cells [45], (4) CFTR and calcium-activated chloride channels are co-localized in the same sweat coil secretory cells [31] (presumably the clear cells) [46]. Previously, it was not possible to distinguish between a specific effect of CFTR loss on cholinergic sweating and a non-specific effect of the reduced health and inactivity of many CF subjects, which reduce sweat rates [47]. Ivacaftor provides the tool to make that distinction. Indeed, for S9, who had a multi-fold increase in C-sweat on ivacaftor, we also detected a small but significant increase in M-sweating on ivacaftor (Fig 4B and Fig 9). It will be important to clarify this point with more extensive experiments, because if M-sweating has a CFTR-dependent component it will result in a slight underestimate of C-sweat when expressed as a percent of WT.

### Summary and conclusions

CF clinical medicine is being transformed by compounds that directly improve CFTR function. Many elegant methods are available to quantify CFTR function *in vitro*, but there is a pressing need for accurate estimates of CFTR function *in vivo*. The present experiments provide quantitative data showing that oral dosing with ivacaftor increases R117H *P*_*O*_. The use of multiple, identified glands and the ability to ratio C- and M-sweat are ideal for n-of-1 studies of CFTR-directed therapeutics. The sweat rate assay complements the standard pilocarpine iontophoresis sweat chloride assay, which is most sensitive in the range of zero~5% CFTR function, a range where the sweat rate assay lacks sensitivity. Conversely, the sweat rate assay is sensitive in higher regions of CFTR function; regions that will become important as modulator efficacy improves. The discordance between the low level of CFTR function assessed by this sweat rate assay and the normal standard sweat chloride test values for S9 provides a cautionary note about over-reliance on the standard sweat test for diagnosis and for assessing the efficacy of CFTR-directed therapies. Precise assessments of ivacaftor’s *in vivo* effects on human CFTR (including WT CFTR) should be useful for many aspects of drug development, including optimizing dosing and assessing drug interactions.
